# Efficacy of surgeon-directed postoperative local injection with an analgesic mixture in posterior fusion surgery for adolescent idiopathic scoliosis

**DOI:** 10.1186/s12891-022-05158-3

**Published:** 2022-03-04

**Authors:** Hiroto Makino, Shoji Seki, Katsuhiko Kamei, Yasuhito Yahara, Yoshiharu Kawaguchi

**Affiliations:** Department of Orthopaedic Surgery, University of Toyama, Faculty of Medicine, 2630 Sugitani, Toyama, Toyama 930-0194 Japan

**Keywords:** Adolescent idiopathic scoliosis, Spinal fusion, Postoperative pain, Opioids, Nonopioid analgesia, Ropivacaine, Epinephrine, Dexamethasone, Nausea and vomiting, Constipation, Patient-controlled analgesia

## Abstract

**Background:**

Severe postsurgical pain in posterior spinal fusion is common. Multimodality analgesia, including opioid-based patient-controlled analgesia (PCA), is commonly used, but opioid-related adverse events such as nausea and vomiting are sometimes a problem. We used a ropivacaine-epinephrine-dexamethasone mixture given as one-time local bilateral submyofascial injections at the operated levels added to conventional multimodality analgesia including PCA for postoperative pain control in one group of patients to confirm whether administration of this mixture reduced postoperative pain and opioid use status post posterior spinal fusion.

**Methods:**

We retrospectively reviewed 67 consecutive patients who had undergone posterior fusion surgery for adolescent idiopathic scoliosis (AIS), 35 of whom were treated with conventional analgesia that consisted mainly of PCA (control group) and 32 of whom were treated with one-time submyofascial injections of a ropivacaine-epinephrine-dexamethasone mixture (submyofascial injection group) added to conventional multimodality analgesia. We compared postsurgical pain levels and the amount of opioid use over the first 48 h after surgery, as well as physical activity levels and adverse events 2 weeks after surgery.

**Results:**

Postsurgical pain quantified by a numeric rating scale (1–10) in the submyofascial injection group was significantly lower than that in the control group. The amount of fentanyl use was significantly less in the submyofascial injection group at 24 h, 48 h, and all subsequent periods after surgery. In addition, Walking Recovery Time (WRT) defined as the number of days until the first event of ambulation was significantly less in the submyofascial injection group (3.3 d vs 4.1 d, *P* = 0.0007)). Laxative use was significantly less in the submyofascial injection group (0.3 times vs 1.3 times, *P* = 0.02).

**Conclusions:**

One-time submyofascial injections at the operated levels with a ropivacaine-epinephrine-dexamethasone mixture after spinal fusion surgery reduced pain, opioid consumption, and opioid-related adverse events. This technique can contribute significantly to postoperative analgesia.

## Background

Adolescent idiopathic scoliosis (AIS) is a relatively common disease. Depending on race, it is found in approximately 0.5–4% of the population [1]. Since the curvature in AIS sometimes progresses, and may cause dysfunction and back pain, bracing of curvatures > 25° is usually undertaken. Surgery is always indicated for curvatures ≥40° [2]. Posterior fusion surgery is the most common procedure for AIS. It is associated with severe postoperative pain [3, 4]. Treatments for this pain include intravenous patient-controlled analgesia (IV-PCA), epidural anesthesia, and acetaminophen and non-steroidal anti-inflammatory drugs (NSAIDs). However, many postoperative patients still experience moderate pain, and some adverse events from the analgesics may occur, especially in the case of opioids, which are associated with postoperative nausea and vomiting (PONV) and constipation. These are associated with longer length of hospital stay and higher cost of care [5]. Some recent studies have shown positive effects of local infiltration of analgesics for postsurgical pain in posterior spinal fusion [6–8].

We developed a one-time local submyofascial injection procedure with a ropivacaine-epinephrine-dexamethasone mixture for postoperative analgesia. This method is easy to apply and combine with other analgesic methods such as IV-PCA. The purpose of the present study was to confirm whether this method reduced postsurgical pain and opioid use.

## Materials and methods

### Data collection

A total of 67 consecutive patients underwent posterior fusion surgery for AIS from January 2016 to March 2019. The electronic health record was used for data collection. From this data source, we extracted information on age, gender, height, weight, range of fusion, Numeric Rating Scale (NRS) of pain after surgery (range; 0–10), the amount of fentanyl used by PCA, walking recovery time (WRT) defined as the number of days until the first event of ambulation, and adverse events that may have been associated with postoperative analgesics including constipation, nausea, surgical site infection, and pseudarthrosis.

### Surgical methods

All patients underwent surgery by a single surgeon (S.S.) at a single hospital. To briefly describe the previously reported surgical procedure [9], surgery was performed under general anaesthesia in the prone position. After pedicle screw insertion, the articular cartilage of the facet joints was removed. A Ponte procedure [10] was performed at three levels near the apex of the curvature. A concave rod (5.5 mm CoCr) was rotated 90° to transform the scoliosis into a thoracic kyphosis or a lumbar lordosis depending on the type of the scoliosis [9]. Then a convex rod was inserted, and the rib hump was reduced using a differential rod-contouring technique followed by segmental direct vertebral rotation of each vertebra [11]. Spinal cord monitoring of motor and somatosensory evoked potentials was performed during surgery.

### Postoperative management

**Fig. 1:**
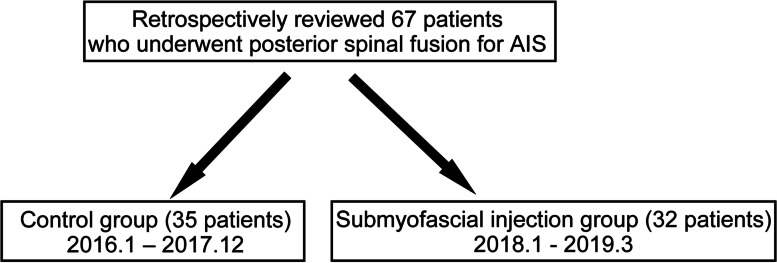
Overview of patients reviewed in this study

After surgery, intravenous patient-controlled anesthesia (IV-PCA) with fentanyl diluted with saline was used in all patients. The lockout time of the PCA pump was 15 min. All patients were allowed to take oral analgesics such as NSAIDs (patients > 15 y) or acetaminophen (patients < 15 y) from next day after surgery. In addition, they were also allowed to use rectal analgesics or intravenous acetaminophen as needed. The submyofascial drain was removed 3 d after surgery and patients were allowed to stand and walk as their pain allowed. Metoclopramide for nausea and laxatives for constipation were used as needed. The patients undergoing surgery between January 2016 and December 2017 were treated with this analgesic protocol (control group), while the patients after January 2018 were additionally treated with a one-time postoperative ropivacaine-epinephrine-dexamethasone mixture injection (submyofascial injection group, Fig. 1).

### Local injection with a ropivacaine-epinephrine-dexamethasone mixture

**Fig. 2:**
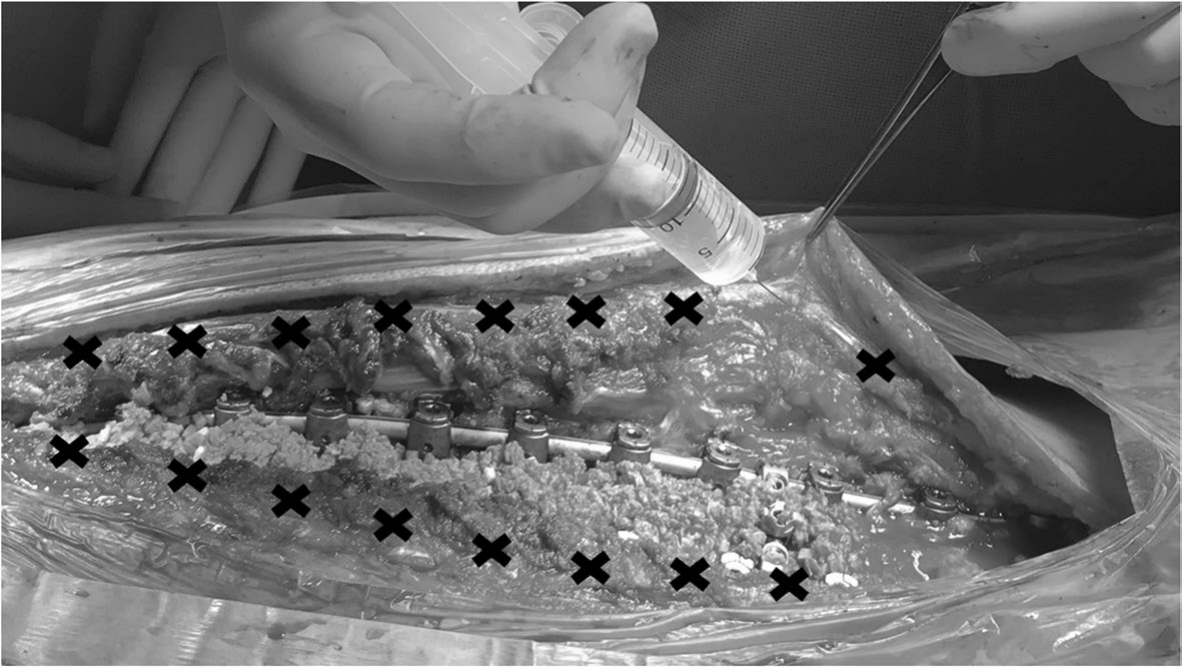
The method of local injection with analgesic mixture. Bilateral infiltration of 1–2 mL of the mixture into the submyofascia at each disc levels

We used a mixture of ropivacaine (3 mg/kg), epinephrine (0.003 mg/kg), and dexamethasone (0.66 mg/kg), diluted up to 50 mL with saline. Before myofascial closure, bilateral 1–2 mL submyofascial injections of the mixture were administered at the operated levels (Fig. 2).

### Statistical analysis

Data are shown as the mean and standard deviation. We used a two-way repeated measures ANOVA followed by the Tukey-Kramer *post-hoc* test for comparative analyses of NRS. Student’s *t*-test was used to compare the demographics and the results of the amounts of opioids needed, the WRT, the number of times an antiemetic was needed, and the number of doses of laxatives that were needed between the control and submyofascial injection groups. *P* values < 0.05 were considered statistically significant. All statistical analysis was done using commercial software (Statcel4, OMS Publishing Inc., Japan).

## Results

Of the 67 patients in total, 35 were in the control group and 32 were in the submyofascial injection group (Fig. 1). There were no significant differences in age, gender, weight, height, body mass index, or number of fusion levels in surgery between the two groups (Table 1).

**Table 1:** The demographics of patients in this study

	Control	Submyofascial injection	P
Number	35	32	
Age (y)	15.0 ± 2.3	15.5 ± 2.6	0.44
Gender (Women %)	32 (91.4%)	27 (84.4%)	0.37
Height (m)	1.57 ± 0.08	1.57 ± 0.07	0.99
Weight (kg)	48.3 ± 9.4	48.7 ± 6.3	0.85
Number of fusion levels	10.4 ± 1.9	9.3 ± 2.3	0.06

### Evaluation of changes in postoperative pain and opioid consumption

**Fig. 3:**
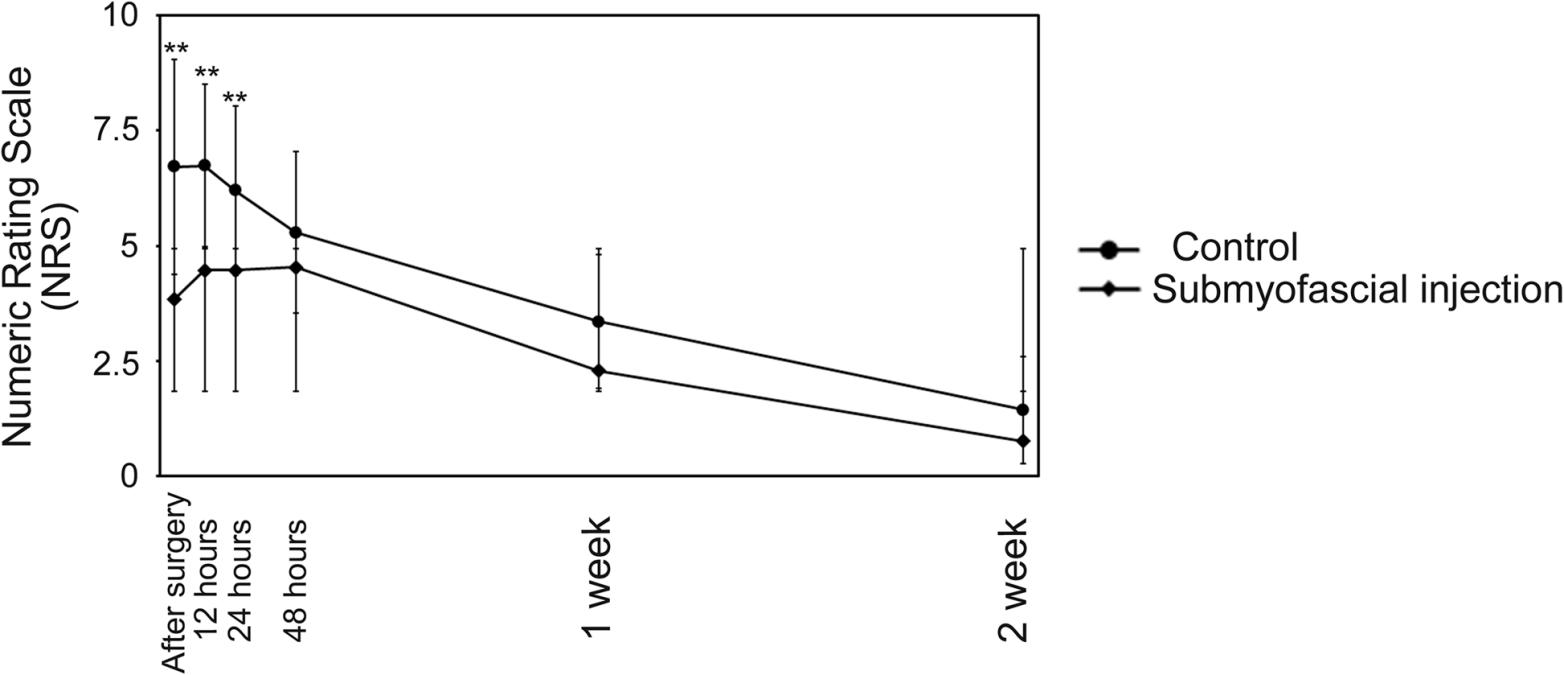
Comparison between the two groups of changes over time in the numeric rating scale (NRS) pain score. ** *P* < 0.01

**Fig. 4:**
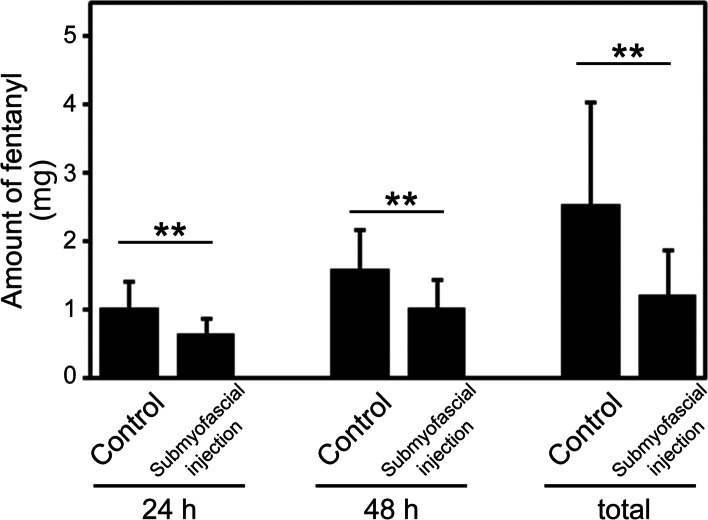
Comparison between the two groups of the amount of fentanyl used at 24, 48 h and in total after surgery. ** *P* < 0.01

Although postoperative pain evaluated by NRS decreased over time in both groups, it was severe in the immediate postoperative period. The NRS values at 0 h, 12 h and 1 d after surgery in the submyofascial injection group were significantly lower than those in the control group. There was no longer a significant difference at ≥2 d (Fig. 3). The amount of fentanyl used was significantly less in the submyofascial injection group than in the control group at 24 h, 48 h, and in total (Fig. 4).

### Comparison of postoperative activity

**Fig. 5:**
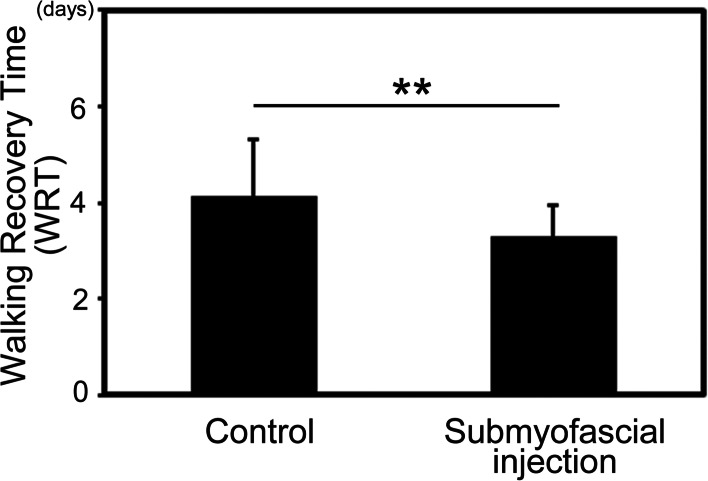
The walking recovery time (WRT) defined as the number of days until the first ambulation after surgery in the two groups. ** *P* < 0.01

The WRT was 3.3 d in the submyofascial injection group, significantly less than the 4.1 d in the control group (Fig. 5, *P* = 0.0007).

### Adverse events and complications after surgery

Of all 67 patients, 33 (49.2%) patients had nausea requiring an antiemetic after surgery. In the control group, 19 (54.2%) patients needed antiemesis, whereas, 14 (43.8%) patients did in submyofascial injection group. This was not significantly different between groups (*P* = 0.26, Table 2).

**Table 2:** Comparison of treatment for adverse events

	Control	Submyofascial injection	P
Use of antiemetic (times)	1.0 ± 1.2	0.69 ± 1.1	0.26
Duration of constipation (days)	5.0 ± 1.8	4.7 ± 1.3	0.43
Use of laxative (times)	1.3 ± 1.7	0.3 ± 0.6	< 0.01

There was no significant difference between groups concerning the recovery of transit materialized by the first defecation (mean 4.9 d after surgery). However, the mean number of times using a laxative was 1.3 times in the control group and 0.3 times in the submyofascial injection group, which was significantly less than in the control group (*P* <  0.01, Table 2).

There were no surgical site infections or neurological deterioration in any patient. There was no local anaesthetic systemic toxicity in the submyofascial injection group. In addition, there were no pseudarthroses following fusion surgery in any cases.

## Discussion

Postsurgical pain after posterior fusion for idiopathic scoliosis is severe, and use of opioids for severe pain may lead to adverse events such as nausea and constipation.

We performed submyofascial injections with a ropivacaine-epinephrine-dexamethasone mixture to reduce postoperative pain and opioid use and found that it significantly reduced postoperative pain on an NRS up to 1 d after surgery. It also significantly reduced opioid use and opioid-related adverse events and shortened the WRT.

The combined use of acetaminophen/NSAIDs for opioid-based postoperative analgesia is widely applied after orthopaedic surgery including spinal fusion. There have been some reports investigating the effect of acetaminophen/NSAIDs on postoperative analgesia, focusing on spinal fusion surgery for scoliosis [12–14]. They showed that acetaminophen/NSAIDs reduced postsurgical pain. However, Hiller et al. [12] reported that acetaminophen reduced postsurgical pain on a visual analogue scale but did not reduced opioid consumption. Analgesia with acetaminophen/NSAIDs alone is usually inadequate, and other analgesic methods have been tried. The most common method of analgesia is IV-PCA with opioids [15], and we have used this method as the basis for postoperative analgesia. While the efficacy of IV-PCA has been widely reported [16], opioid-related adverse events such as PONV are of concern [17].

To achieve reductions in both postsurgical pain and opioid consumption, some methods combined with IV-PCA have been tried. First, epidural analgesia with/without IV-PCA is also widely used for postsurgical pain in AIS undergoing posterior spinal fusion. One meta-analysis [18] showed that epidural analgesia had a better postoperative effect than IV-PCA. However, whether epidural analgesia reduced opioid-related adverse events such as PONV is still unknown [3, 19, 20]. Moreover, there have been concerns about adverse events such as respiratory depression; neurological changes, which are of the greatest concern to surgeons; oversedation; and catheter problems [21]. Intrathecal administration of opioids has been reported [22, 23]. Although this method contributes to analgesia and reduction of opioid use in the early postoperative period, respiratory depression can still be a problem [22]. Continuous wound infiltration has been recently used for postoperative analgesia [6–8]. This method is useful in reducing postoperative pain and opioid use, and although no serious complications have been reported, there have been concerns about the potential risk of surgical site infection associated with catheter placement [3]. We hypothesized that a single intraoperative infiltration of analgesics could eliminate this potential risk of infection and provide pain relief. In fact, local infiltration with analgesics for postsurgical pain is widely applied, especially in total knee arthroplasty and total hip arthroplasty, and its efficacy in reducing postsurgical pain and opioid consumption has been confirmed [24, 25]. One-time submyofascial injection of a ropivacaine-epinephrine-dexamethasone mixture was useful for postsurgical pain in this study. The reasons for choosing the submyofascia as the injection site are that pain receptors are present in this layer [26] and that the posteromedial branch of the spinal nerve passes through the fascia. Recently, the analgesic effect of local injection in the submyofascia of the erector spinae has been reported [27].

This study has some limitations. First, this study was retrospective, and not a randomized controlled trial. A single surgeon at a single facility performed all the operations, so the variations in pain due to different surgical techniques were avoided. Second, the best mixture, concentration of local analgesics, and administration site (subcutaneous or submyofascial) are unknown. The effect of dexamethasone on postsurgical pain after posterior spinal fusion has been reported recently [28].

## Conclusion

One-time postoperative local submyofascial injections with a ropivacaine-epinephrine-dexamethasone mixture reduced pain and the need for opioids after posterior spinal fusion. Injection of this mixture may be a useful adjunct to conventional analgesia in posterior spinal fusion for AIS.

## Data Availability

The datasets used and/or analysed during the current study are not available publically due to privacy, but are available from the corresponding author on reasonable request.

## Abbreviations

AIS: Adolescent idiopathic scoliosis
PCA: Patient-controlled analgesia
WRT: Walking recovery time
IV-PCA: Intravenous patient-controlled analgesia
NSAIDs: Non-steroidal antiinflammatory drugs
PONV: Postoperative nausea and vomiting
NRS: Numeric rating scale
